# Dental Diagnosis

**Published:** 1884-10

**Authors:** A. T. Bigelow

**Affiliations:** Bismark, Dakota.


ARTICLE VI.

### DENTAL DIAGNOSIS.

BY A. T. BIGELOW, D. D. S., BISMARK, DAKOTA.

To every one who considers the matter, it must be evident that this is an important branch of dental pathology.

The scope of this paper will be limited to the considera- tion of a few points, deemed essential, mostly the deductions of actual observation and experience.

Ætiology will not be touched on, but rather the ascertain ment of actual conditions on inspection, as a basis for intelligent professional service.

The face of every tooth should be presented, directly o by reflected image, to the eye, especially the approxima surfaces whenever there is reason, from general indication, to suspect decay.

Stress is laid on this, on account of the approximal surfaces being less accessible, and as being the part most commonly neglected.

Direct separations by wedging, if the teeth are not firm in sockets, will afford requisite space, or a section of French tubing number ten, employed where the first method is not practicable.

Given an educated eye, a good plain five eights inch mirror, slightly heated, several delicate exploring instruments of suitable construction, and "ocular proof" may be had of the encroachment of the greatest enemy, caries. On a suitable diagram should be noted the defects revealed, beginning with the right dens sapientiæ as number one, and ending with its antagonist as number thirty-two.

This will afford a comprehensive idea of the general condition of the organs and their needs.

If a complete examination is not desired, but only attention to immediately necessitous cases, these may be easily detected on mesial and distal surfaces of bicuspids and molars by drying with a hot air syringe, and observing the discoloration, or opaqueness, ranging from pale yellow to blueish black.

In all cases where decay has progressed extensively and the patient has felt marked discomfort, or pain, very nice discrimination will need to be exercised to effect a sound diagnosis. Would first ascertain for how long time and to what extent disagreeable sensations have been experienced. If of recent origin and no more than an occasional "twinge," capping at once would be indicated.

If the trouble has existed for some weeks the pain at times lancinating, with intervals of quiet, there is considerable inflammation, and soothing applications should be made and the air excluded for a time, before permanent stopping is effected. In more serious cases where the sharp and intermittent have merged into constant grinding pains, there is much inflammation .with congestion, probably involving the whole of the ganglion and possible extending into one or more of the canals. Very likely on removal of the detritus there will appear a protrusion of the pulp into the cavity of decay. If without suppuration would resort to a little blood-letting by acupuncture to relieve distended vessels, applying topical dressing. Should the case yield kindly and the inflammation subside without suppuration, the operation of capping and filling may eventually be performed with a good prospect of success. Have never tried Dr. Allport's method of incising the protuberant pulp to accomplish the same object, the needle lance operation being less heroic but equally effectual in results, the enlargement usually contracting to normal size on depletion of blood vessels, in a few days.

If the more advanced stage has been reached, and partial sepsis occurred, would deem capping contra-indicated. I have dwelt at some length on the indications favorably and otherwise for saving the life of the tooth, having been frequently called upon by patients in transit to relieve them from severe suffering. On minute examination have found too often, for the honor of the profession, fillings inserted on devitalized pulps, or that the operation of capping had been so inconsiderately, or unskillfully performed that the death of the pulp ensued.

Recently service was rendered a patient who said her teeth were pronounced in good condition by her family dentist only a short time before she left for the West. The name given is not unknown to the profession, and the gold work exhibited was exceptionally fine. Only a glance was needed to disclose the tell tale opaqueness on mesial surface of first superior molar, and excavation developed a large cavity that must have had its inception more than a twelve month previously. Was it culpable negligence or want of apprehension of pathological indications? This is cited as a typical case to show the necessity of systematic practice that our clientage may not suffer, from the defective faculty being faulty, where the manipulative ability is excellent. There are other and less common dental affections that require much skill and experience in determing their nature, in some instances baffling all known means of ascertainment. Will offer a few selected from my case book, in illustration. Patient complained of a slight unpleasantness in one of the superior incisors. No external defect appeared. The color of left central was a shade darker than that of the adjoining teeth, but with no more variation than is often seen between incisors and cuspidati. No marked sensation produced on tapping with an instrument. In such a case the phonetic or percussive method may be adopted in detect a necrosed tooth, similar to that employed in diagnosing diseases of the thorax or abdomen.

The most available agent to employ is the ivory handle of a mouth mirror. It should be held between the thumb and second finger of right hand and gently tapped with index finger, applying the blow to the labial surface. The sound elicited from a normal tooth will be found clear and resonant, while the necrosed organ yields a sound perceptibly lower in pitch and more muffled or obscure. These distinctions have proved constant so far as I have yet experimented and may be relied upon in determining those infrequent cases where from a blow or some unknown cause, the pulp has lost its vitality. The proper employment of this mode necessitates some degree of skill in application, and the possession of a musical ear, capable of detecting minute variations in quality of sound.

A young married lady consulted me on account of severe pain located in first left superior molar, for which, after the most careful scrutiny, I was unable to assign any adequate cause. There was a small gold filling on grinding, and a somewhat larger one on anterior approximal surface, but not suspect that the latter encroached upon the pulp. There seemed to be no symptoms of exostosis and the general health of patient precluded any probability of the cause being reflex or sympathetic. In my perplexity I had recourse to the Micawber plan, hoping something might

"turn up" to help me out of the dilemma. The only clew afforded was that in treating the corresponding molar, about six months previously, a small calcification had been discovered in the pulp after its removal, and this seemed a good opportunity to test the bilateral theory. Not wishing, however, to make a serious error in treatment, I removed the larger filling, applied a pellet of cotton saturated with pure wood creasote, painted the gums with aconite and iodine, and requested the patient to call next day. She reported early in the morning, having suffered considerably during the night, and desired the tooth extracted. This I declined to do, but made the usual application to destroy pulp, and on the second day thereafter had the satisfaction of finding in distal portion of ganglionic cavity a large calcification, having somewhat the form of an eclipse, measuring in length twelve one hundredths, and in the short diameter, six and one half one hundredths of an inch. Viewed superficially with a low power microscope, it presents more the appearance of cementum than of enamel or dentine. It is presumably of the same constitution as the so called secondary dentine.

I will now give the history of a unique case. A healthy looking young lady, about twenty years of age, visited my office on account of severe pain, located in first and second right superior molars. Careful examination showed no cause, in fact her dentine was exceptionally perfect in form and structure, no caries being present. I had much difficulty in persuading her to delay the extraction for a day or two ; meantime topical application was made, more to gain time than with any expectation of amelioration. On the second day there was observed, over the seat of pain, a slight swelling, accompanied by redness and inflammation. Being satisfied it was a disease, not having a strictly dental origin, the patient was recommended to consult a physician. The doctor advised with, pronounced it erysipelas. The case was successfully treated and she had no further trouble with her teeth.

[TO BE CONTINUED.]

